# Bayesian Optimal Designs for Multi-Arm Multi-Stage Phase II Randomized Clinical Trials with Multiple Endpoints

**DOI:** 10.1080/19466315.2024.2344543

**Published:** 2024-05-17

**Authors:** Guillaume Mulier, Sylvie Chevret, Ruitao Lin, Lucie Biard

**Affiliations:** a INSERM U1153, Epidemiology and Clinical Statistics for Tumor, Respiratory, and Resuscitation Assessments (ECSTRRA) Team, Paris, France; b Department of Biostatistics, MD Anderson Cancer Center, Houston, TX

**Keywords:** Bayes, Multi-arm, Oncology, Phase II, Randomization

## Abstract

There is a growing need to evaluate of multiple competing drugs in phase II trials where the number of patients is often limited, and simultaneous assessment of both efficacy and toxicity is crucial. To avoid the waste of research resources, it is indeed more efficient to screen multiple drugs at once in a platform phase II setting. We aim to adapt the Bayesian optimal phase II (BOP2) design to multi-arm trials for both uncontrolled and controlled settings. The binary efficacy and toxicity endpoints are modeled by a Dirichlet distribution as a vector of four outcomes. Posterior marginal distributions at each analysis are used to derive the monitoring threshold that varies during the trial. We control the family-wise Type I error rate for multiple comparison against a common reference value or a shared control. We conduct simulation studies under both uncontrolled and controlled settings to evaluate the operating characteristics of the proposed design. Our simulations demonstrate that the design exhibits better operating characteristics compared to a design using a constant threshold and is less sensitive to changes in accrual rate relative to what was planned. The design had promising operating characteristics and could be used in phase II oncology clinical trials for evaluating multiple drugs at a time.

## 1. Introduction

In oncology, phase II trials have long employed single-arm designs to assess the efficacy of new drugs. However, over the past few decades, the development of drugs in oncology, particularly immunotherapy agents, has been on the rise, creating a growing need for enhanced evaluation of their toxicity and efficacy. Indeed, the evaluation of multiple new treatments separately poses challenges in estimating the relative effect of each treatment, due to “treatment-trial” confounding (Estey and Thall 2003): Trials for different drugs are often designed using distinct eligibility criteria, standards of care, and outcome measures, which can confound the estimation of the relative treatment effect. Moreover, this one-at-a-time evaluation of the treatments in early phases appears to lack specificity in detecting effective drugs, as evidenced by the reported low success rates in phase II and III trials and subsequent market approvals of cancer therapeutics (Sutter and Lamotta 2011).

With the aim of avoiding confounding and shortening the duration of drug development, platform trials that evaluate multiple drugs (either simultaneously or at different times) have been proposed (Yu, Hubbard-Lucey, and Tang 2019; Franklin et al. 2022). Platform trials extend the concept of randomized phase II trials that include a control group, a concept first proposed in the early 90s (Simon, Thall, and Ellenberg 1994). They offer well-documented advantages of the randomized trials over single-arm trials (Sharma, Stadler, and Ratain 2011; Wason, Stecher, and Mander 2014; Hobbs, Chen, and Lee 2018). One notable benefit is the ability to screen several candidate treatments allocated through randomization and identify the most promising ones. More specifically, multi-arm multi-stage (MAMS) designs (Hobbs, Chen, and Lee 2018) establish early decision rules based on sequential analyses of the effect of multiple treatments compared to the control arm. These designs aim to control the false positive rate for the entire trial, not just for each arm separately (Jaki, Pallmann, and Magirr 2019). Additionally, MAMS designs address both ethical and economic concerns by allowing for the early termination of treatments with evidence of futility and/or excessive toxicity as well as the early graduation of promising treatments.

The literature proposing Bayesian approaches for adaptive platform trials is expanding (Berry 2006; Berger, Wang, and Shen 2014; Ryan et al. 2019). Among these approaches, the Bayesian Optimal design for Phase II clinical trial (BOP2) allows for evaluation of multiple endpoints sequentially, although it is restricted to the single-arm setting (Zhou, Lee, and Yuan 2017). In brief, the BOP2 design aims to assess a new treatment in comparison to reference values of m binary outcome measures. The resulting $2^{m}$ distinct and mutually exclusive outcomes define a multinomial random variable. Inference on the model parameters is conducted within a Bayesian framework, using a Dirichlet prior, chosen for its conjugacy with the multinomial distribution. Due to the aggregation properties of the Dirichlet distribution, the marginal Beta distributions of each of the m binary outcomes can be computed easily, allowing the derivation of stopping rules for futility or excessive toxicity(Zhou, Lee, and Yuan 2017).

We aimed to extend the BOP2 design to accommodate multiple treatment arms (or multiple doses of the same treatment), all evaluated concurrently for both efficacy and toxicity within the same trial. To illustrate this extension, we retrospectively applied the proposed design to the AZA-PLUS trial (NCT01342692), which was conducted in patients with high risk myelodysplastic syndrom (MDS) (Ades et al. 2018).

The article is organized as follows: First, we present the models and design algorithms. Next, we conduct a simulation study to assess the performance of the designs. We then illustrate the proposed designs retrospectively using the AZA-PLUS trial (NCT01342692). Finally, we provide a discussion.

## 2. Motivating Example: AZA-PLUS Trial

In the treatment of adult patients with high risk (defined by an intermediate-2 or high IPSS score) myelodysplastic syndrom, the AZA-PLUS trial (NCT01342692) aimed to evaluate whether the efficacy and toxicity of the standard-of-care azacitidine could be improved by adding a new drug (Ades et al. 2018). Initially, the trial was first planned to assess the combination of azacitidine with lenalidomide or valproic acid, and later with idarubicine, using the Jung’s two-stage design(Jung 2008). The trial was designed with a Type I error rate of 0.15 and a Type II error rate of 0.20. It was scheduled to recruit 80 patients in each arm, with an interim analysis conducted after 40 patients per arm.

In total, 322 patients were enrolled from June 2011 to July 2017 across 37 participating centers. The treatment arms included 81 patients receiving azacytidine (AZA), 80 receiving AZA + valproic acid (AZA+VPA), 80 receiving AZA + lenalidomide (AZA+LEN), and 81 receiving AZA+idarubicin (AZA+IDA). Unfortunately, there was no evidence of any benefit from any of the combinations.

Our focus was on the three-arm randomized trial, which compared the control (AZA, n=81) against both AZA+LEN (n=80) and AZA+VPA (n=80). We considered two binary outcome measures: the efficacy endpoint was the overall response rate (ORR), defined by the achievement of complete, partial, or medullary remission, and hematological improvement after 6 treatment cycles (a 6-month period). The toxicity endpoint was defined as treatment discontinuation due any reason other than progression or relapse. Based on the terminal analysis of the 241 enrolled patients, the pooled ORR was estimated at 41.1%, with arm-specific estimates of 42.0%, 41.2%, and 40.0% in the AZA, AZA+VPA, and AZA+LEN, respectively. The toxicity rates ranged from 59.3% in the AZA arm to 65.0% in the AZA+VPA arm and 67.5% in the AZA+LEN arm. To assess whether the generalized BOP2 design for multi-arm multi-stage trials could have allowed us to interrupt the trial earlier, we retrospectively applied this proposed design to the trial data.

## 3. Methods

We extended the BOP2 design to a multi-arm multi-stage trial, where patients are randomized to K experimental arms in an uncontrolled setting or to K experimental arms plus a control arm in a controlled setting. For simplicity, we assumed balanced randomization across the investigational arms. We considered only two binary outcomes, $\left(Y_{T},Y_{E}\right)$, where $Y_{T}=1$ indicates toxicity and 0 otherwise, and $Y_{E}=1$ indicates efficacy and 0 otherwise. It’s worth noting that the design can be readily extended to handle unequal randomization, adaptive randomization schemes, or more than two endpoints.

Let $Yk\;=\;(Y_{k,T},Y_{k,E})$ represent the co-primary toxicity and efficacy endpoints observed in arm k=0,…,K (with k=0 denoting the control arm in a controlled setting). Thus, Yk follows a multinomial distribution with probability vector ($\theta_{k,TE}$, $\theta_{k,\bar{T}E}$, $\theta_{k,T\bar{E}}$, $\theta_{k,\bar{TE}}$). These probabilities correspond to the four possible events: TE for efficacy and toxicity, $\bar{T}E$ for efficacy and no toxicity, $T\bar{E}$ for toxicity without efficacy, and $\bar{TE}$ for no toxicity nor efficacy. The prior of those parameters was defined by a Dirichlet ($\pi_{0,TE}$, $\pi_{0,\bar{T}E}$, $\pi_{0,T\bar{E}}$, $\pi_{0,\bar{TE}}$) with $\pi_{0,}$. denoting the probabilities under the inefficacy/toxicity hypothesis. We set $\pi_{0,TE}+\pi_{0,\bar{T}E}+\pi_{0,T\bar{E}}+\pi_{0,\bar{TE}}=1$ to ensure that the prior effective sample size is 1. Due to the properties of the Dirichlet distribution, the marginal prior distributions of efficacy and toxicity outcomes in arm k, $p_{k,E}$, and $p_{k,T}$ can be easily derived as $\text{Beta}(\pi_{0,TE}+\pi_{0,\bar{T}E},\pi_{0,T\bar{E}}+\pi_{0,\bar{TE}})$ and $\text{Beta}(\pi_{0,TE}+\pi_{0,T\bar{E}},\pi_{0,\bar{T}E}+\pi_{0,\bar{TE}})$, respectively. Due to conjugacy, the posterior distribution of $\theta_{k}\;=\;(\pi_{0,TE},\pi_{0,\bar{T}E},\pi_{0,T\bar{E}},\pi_{0,\bar{TE}})$ also follows a Dirichlet distribution. Therefore, the posterior distributions of $p_{k,E}$ or $p_{k,T}$ follow Beta distributions, which can be easily obtained based on the numbers of efficacy and toxicity outcomes, $x_{k,E}$ and $x_{k,T}$, among the $n_{k}$ enrolled patients in arm k, respectively. More specifically, let $D_{n,k}=\{x_{k,E},x_{k,T},n_{k}\}$ denote the observed data in arm k, then we have $p_{k,E}|D_{n,k}\;∼\;\text{Beta}(\pi_{0,TE}+\pi_{0,\bar{T}E}+x_{k,E},\pi_{0,T\bar{E}}+\pi_{0,\bar{TE}}+n_{k}-x_{k,E})$, and $p_{k,T}|D_{n,k}∼\text{Beta}(\pi_{0,TE}+\pi_{0,T\bar{E}}+x_{k,T},\pi_{0,\bar{T}E}+\pi_{0,\bar{TE}}+n_{k}-x_{k,T})$.

Similar to the original BOP2 design, we considered I interim analyses, defining early futility and toxicity stopping rules. The maximum sample size for each arm was set at $N_{k}=\sum_{i}n_{i,k}$ patients, where $n_{i,k}$ denotes the number of patients enrolled in arm k during the ith interim analysis, i=1,…,I. We first derived the generalized BOP2 design in the uncontrolled setting, where each treatment was compared with prespecified historical efficacy and toxicity rates. Subsequently, we then adapted the BOP2 design to the controlled setting, in which each treatment was compared to a common control.

### 3.1. Uncontrolled Setting

Define prespecified targeted efficacy and toxicity rates as $\phi_{E}$ and $\phi_{T}$, respectively. These values are elicited based on expert opinions and historical data. Thus, the hypotheses for each treatment arm k are as follows:

$${\text{Futility or Toxicity}}_{k}\;:\;\;\;\;\;\;\;p_{k,E}\leq \phi_{E}\;\text{or}\;p_{k,T}>\phi_{T}\;{\text{Efficacy and Non toxicity}}_{k}\;:\;\;p_{k,E}>\phi_{E}\;\text{and}\;p_{k,T}\leq \phi_{T}.$$

At each interim analysis, which is conducted once $n_{k}$ patients have been treated and assessed for both efficacy and toxicity endpoints, the stopping decision for futility and/or toxicity are made using the following rules:

{P(pk,E≤ϕE∣Dn,k)>CnP(pk,T>ϕT∣Dn,k)>Cn}

where $P(\cdot |D_{n,k})$ is derived from the posterior Beta distribution of the marginal probability of $p_{k,E}$ or $p_{k,T}$, and $0<C_{n}<1$ is the decision threshold (as defined in Section 3.3).

### 3.2. Controlled Setting

In the setting of a controlled trial, which includes a common control arm (k=0), the hypotheses for each experimental arm k>0 are as follows:

$${\text{Futility or Toxicity}}_{k}^{c}\;:\;\;\;\;\;\;\;p_{k,E}\leq p_{0,E}\;\text{or}\;p_{k,T}>p_{0,T}\;{\text{Efficacy and Non toxicity}}_{k}^{c}\;:\;\;p_{k,E}>p_{0,E}\;\text{and}\;p_{k,T}\leq p_{0,T}.$$

The two stopping rules to halt arm k (k>0) for futility and/or toxicity are as follows:

{P(pk,E≤p0,E∣Dn)>CnP(pk,T>p0,T∣Dn)>Cn}

where $D_{n}\;=\;\{x_{k,E},x_{0,E},x_{k,T},x_{0,T},n_{k},n_{0}\}$ is the observed data at the interim analysis for arm k, k=0,…,K, and $C_{n}$ is the common decision threshold as defined in Section 3.3.

Computations of the above two probabilities are carried out through integration, as follows (Jacob et al. 2016; Hobbs, Chen, and Lee 2018):

$$P(p_{k,.}>p_{0,.}|D_{n})=\int_{0}^{1}(1-F(p|D_{n,k}))f(p|D_{n,0})dp$$

where F(⋅) is the Beta cumulative distribution function and f(⋅) is the Beta density function.

### 3.3. Choice of the Decision Threshold

When planning the multi-arm trial, the hypotheses regarding the multinomial distribution of $Y_{k}$. for the efficacy and toxicity of the new treatment, as well as the maximum acceptable Family-Wise Error Rate (FWER), are first elicited with the clinicians. Following the standard BOP2 design by Zhou, Lee, and Yuan (2017), the null hypothesis $H_{0}$ corresponds to an inadmissible treatment (ineffective and overly toxic), while the alternative hypothesis $H_{1}$ corresponds to a promising treatment (effective and not excessively toxic). Additional discussions on the choice of the null hypothesis for testing co-primary toxicity and efficacy endpoints are provided at the end of this section.

Unlike a single arm trial, we must address the presence of K distinct experimental arms when optimizing the decision threshold. In this context, the FWER is defined as the proportion of claiming an inadmissible arm promising, which means concluding the efficacy and acceptable toxicity of at least one experimental arm, while all these arms are inefficacious and toxic (i.e., the global null hypothesis, where each arm is at $H_{0}$). The (least) power is defined as the proportion of concluding the efficacy and acceptable toxicity of the efficacious and safe arm when that arm is the only efficacious and safe arm, and all others are inefficacious and toxic. This scenario is also referred to as the Least Favourable Configuration (LFC), where only one arm is at $H_{1}$ and all others are at $H_{0}$)(Thall, Simon, and Ellenberg 1989; Jaki, Pallmann, and Magirr 2019).

To define the decision boundaries, we first considered the same threshold function as the original BOP2 design (Zhou, Lee, and Yuan 2017). This function is represented as $C_{n}^{s}=1-\lambda {\left(\frac{n}{N}\right)}^{\gamma }$, where λ(0<λ<1) and γ are positive design parameters. In cases where there is only one experimental arm, the values of λ and γ can be optimized by grid search to control the false positive rate and maximize power in both uncontrolled and controlled settings, respectively (Zhou, Lee, and Yuan 2017; Zhao et al. 2023). We further defined γ such that γ≤1, ensuring a convex shape for the decision boundary. The shape of the threshold function reflects the principle that early stopping based on sparse data should be avoided, and the rules become less stringent as more data accumulates over the trial (Jennison and Turnbull 1999).

We then defined a threshold specifically designed for the multi-arm trial, denoted thereafter as $C_{n}^{m}$. We employed the same threshold function, but the design parameters (λ, γ) used to define $C_{n}^{m}$ were optimized through simulation via a grid search that accounts for multiple experimental arms. Among all possible pairs of (λ, γ) satisfying the prespecified FWER constraint (Magirr, Jaki, and Whitehead 2012; Bratton et al. 2016; Jaki, Pallmann, and Magirr 2019) under the global null hypothesis, we selected the one that maximizes power under the LFC. The grid search can be performed based on simulations, see more details in sec. 2.3 of Zhou, Lee, and Yuan (2017). It’s worth noting that as the cutoff function was optimized under the global null and depends only on the sample size, this approach also maintained the arm-specific Type I error rate.

Alternatively, another decision threshold, $C_{n}^{m,a}$, has been proposed, and it depends on the number a of ongoing active experimental arms (those still open to patient accrual) at the time of the interim analysis. It is defined as

$$C_{n}^{m,a}=1-\left(\frac{\eta -\lambda }{\eta }\right){\left(\frac{n}{N}\right)}^{\gamma }$$

where η=K+1−a. This correction relative to a leads to a less stringent threshold, particularly when multiple arms are truly promising as the threshold function is an increasing function of a. To optimize the parameters λ and γ, mimicking the procedure used for optimizing $C_{n}^{m}$, we aimed to control the FWER under the global null and maximize power under LFC. However, due to the construction of $C_{n}^{m,a}$, merely controlling the FWER is insufficient to maintain a desired arm-specific Type I error rate, especially when only one experimental arm is unpromising while the rest are promising (because many arms including the unpromising one can pass the monitoring criteria). To address this issue, there are two approaches. The first approach is to simultaneously control the FWER under the global null and the arm-specific Type I error rate under the scenario where only one arm is unpromising. However, due to the additional constraint in terms of the arm-specific Type I error rate, this approach requires a larger parameter optimization search within a well-defined search region, making it time-consuming. The second approach is more straightforward. By taking the final threshold as the minimum between $C_{n}^{m,a}$ and the single experimental arm-based $C_{n}^{s}$ of Zhou, Lee, and Yuan (2017), it is trivial to demonstrate that the design based on $\left(C_{n}^{m,a},C_{n}^{s}\right)$ can not only maintain the FWER but also control the arm-specific Type I error rate. We chose the latter approach as it requires less computational resources.

Lastly, due to the formulation of the null hypothesis $H_{0}$ (i.e., treatment being ineffective and overly toxic), there is a possibility that the optimal multi-arm design may not maintain tight control over the false positive rate, especially when one of the co-primary endpoints is not met—either the treatment is safe but ineffective, or effective yet overly toxic. This issue is evident in scenarios 6 and 7 of Tables 1 and 2, where the arm-specific Type I error rate or the FWER may exceed the nominal level. To address potential concerns, a more rigorous calibration process involving two null hypotheses, $H_{01}$ and $H_{02}$, could be implemented. $H_{01}$ would consider the treatment as safe but ineffective, whereas $H_{02}$ would view it as effective but overly toxic. The initial step of the calibration process as outlined above could then be extended to identify all feasible (λ, γ) pairs meeting the predefined FWER constraints under both $H_{01}$ and $H_{02}$ ( as well as the arm-specific Type I error constraints for the $C_{n}^{m,a}$ approach). This method, while potentially being less powerful, offers improved control over false positives.

## 4. Simulation Study

### 4.1. Simulation Settings

We conducted various simulation studies to assess the operating characteristics of the proposed generalized BOP2 design in both uncontrolled and controlled settings. A total of K=3 experimental treatment arms were considered, with a maximum sample size of n=60 patients in each group. To assess the proposed designs, we considered the uncontrolled (3 arms) and controlled (thus, with 4 arms) settings, separately, resulting in a maximum total number of included patients of N=180 and N=240, respectively. Three interim analyses were planned to be performed, when every 15 additional patients have been enrolled in each arm, plus the terminal analysis. Of note, we also examined various sample sizes. As expected, in the case of a smaller planned sample size (e.g., 30 patients planned per arm with interim analyses at 10 and 20), power decreased under fixed scenarios. Desirable performance could be achieved with a larger planned effect size, in both uncontrolled and controlled settings (data not shown). All arms were assumed to be of equal size in the main simulation study. Sensitivity analysis, based on different accrual rates among the various arms, was also conducted, as shown below.

A total of 13 different scenarios were constructed similarly for both uncontrolled and controlled settings (see Supplementary Table S1). These scenarios were derived from two real clinical trials: one evaluating the efficacy and safety of lenalidomide associated with rituximab in the treatment of recurrent non-follicular lymphoma (Sacchi et al. 2016) in an uncontrolled setting, and the second comparing TAS-102, a nucleoside analogue, and topotecan/amrubicin for the treatment of refractory small cell lung cancer in a controlled setting (Scagliotti et al. 2016). These motivating examples allowed us to derive realistic hypotheses of efficacy and toxicity targets. In both uncontrolled and controlled settings, scenario 1 corresponded to the global null hypothesis $H_{0}$, while scenario 2 represented the LFC. Scenarios 6, 7, 10, and 11 explored cases of undesirable treatments, including scenarios with no efficacy and toxicity, efficacy and toxicity, and no efficacy and no toxicity. Scenarios 5 and 8 involved treatments with more efficacy or less toxicity than expected. Finally, Scenarios 12 and 13 illustrated treatments with intermediate efficacy and toxicity.

**Figure 1.**
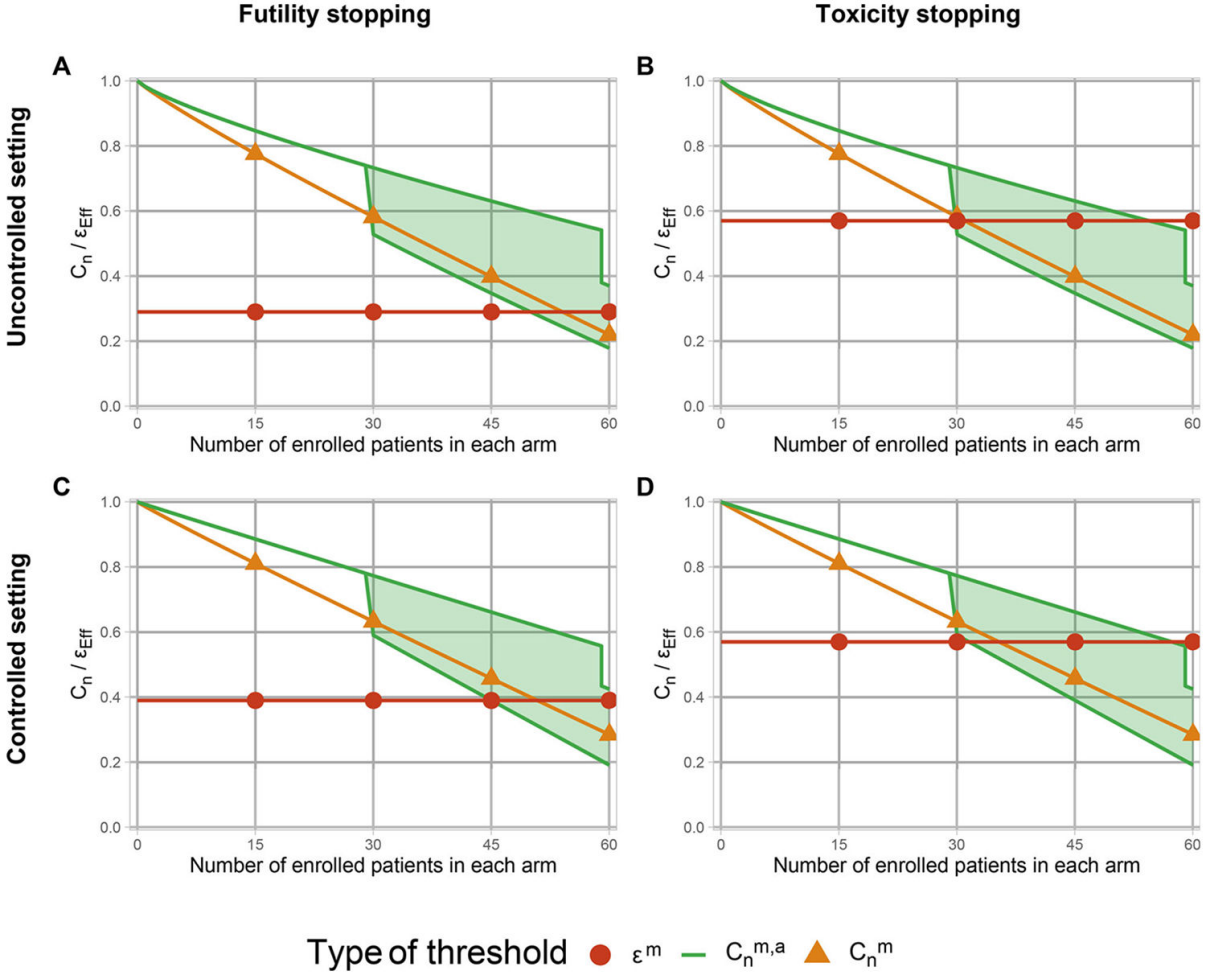
Decision thresholds used during the trial at the interim and terminal analyses, either for futility (A, C) or (over-)toxicity (B, D), based on the uncontrolled design (plots A and B) or the controlled design (plots C and D). $C_{n}^{m}$ stands for the threshold in the same form as BOP2 applied in a multi-arm setting, $C_{n}^{m,a}$ represents the multi-arm threshold dependent on the number of remaining ongoing arms, while the ϵ is the multi-arm constant threshold maintained throughout the trial.

We compared the performances of the proposed decision thresholds ($C_{n}^{m}$, $C_{n}^{m,a}$) with Thall, Simon, and Estey’s approach (Thall, Simon, and Estey 1995), which is similar to the approach of Hobbs, Chen, and Lee (2018), using constant boundaries applied to a multi-arm trial, denoted hereafter $\epsilon^{m}$, and original single experimental arm-based BOP2 threshold $C_{n}^{s}$. All decision thresholds ($C_{n}^{m}$, $C_{n}^{s}$, $C_{n}^{m,a}$ and $\epsilon^{m}$) were computed as described in Section 3.3, using the following hypotheses: for the uncontrolled design $H_{0}$: $\theta_{k}=(0.15,0.30,0.15,0.40)$ and $H_{1}$: $\theta_{k}=(0.18,0.42,0.02,0.38)$ with prespecified targeted efficacy and toxicity rates $\phi_{T}\;=\;0.30$ and $\phi_{E}\;=\;0.45$; and for the controlled design $H_{0}$: $\theta_{k}\;=\;(0.30,0.30,0.10,0.30)$ and $H_{1}$: $\theta_{k}\;=\;(0.25,0.50,0.05,0.20)$ (Figure 1). Decision thresholds were optimized to maximize power while controlling the FWER at 10%.

The prior distributions of the designs were set to reflect the null hypotheses ($H_{0}$) as described in Section 3, resulting in a so-called “skeptical” prior approach (Spiegelhalter, Abrams, and Myles 2004). Consistent with the two real trials that were used to define realistic scenarios, the prior was set to Dir(0.15,0.30,0.15,0.40) for the uncontrolled setting and to Dir(0.30,0.30,0.10,0.30) for the controlled design.

For each scenario, both in the uncontrolled and controlled settings, we conducted 10,000 independent repetitions of each trial, each of K=3 experimental arms. We computed various performance metrics, including the percentage of selection for both efficacy and non toxicity, both globally and for each arm separately, the percentage of correct selection, the empirical FWER of the designs (under the null scenario 1) and the power (under the LFC of scenario 2). Additionally, we calculated the percentage of early stopping, defined as any stopping decision regarding arm k before the terminal analysis, and recorded the reason of the stopping decision (toxicity or futility). It’s important to note that the number of repetitions was determined to ensure a Monte Carlo standard error of 0.003 for a 0.1 Type I error rate and 0.004 for a power value of 0.8, following previous work (Koehler, Brown, and Haneuse 2009; Morris, White, and Crowther 2019).

Results for the uncontrolled setting are reported in Table 1; and results for the controlled setting are provided in Table 2.

We conducted additional analyses to assess the robustness of our results concerning the total sample size, accrual rate, and imbalanced sample sizes across the arms during interim analyses. First, to simulate a lower accrual than expected, we reduced the maximum sample size N in each arm, ranging from 20 to 60 while using the threshold optimized for 60 patients per arm. Interim analyses were performed every N∕4 patients in each arm. It’s important to note that the thresholds were optimized based on a prespecified sample size of 60, rather than the actual sample size, to mimic a real-world scenario where the accrual rate is slower than anticipated. Secondly, to simulate fluctuations in the accrual rate, we performed interim analyses after every $60\times {\left(\frac{j}{4}\right)}^{\psi }$ enrolled patients, with j representing the jth interim analysis and ψ varying from 0.25 to 1.75. A value of ψ=1 represented the planned accrual, with 15 patients recruited in each arm between analyses. Lower values of ψ corresponded to a fast accrual rate at the beginning, while higher values indicated a slower accrual rate at the beginning. Lastly, to assess impact of imbalances in the number of patients recruited at each interim analysis, we conducted interim analyses after the recruitment of 15+{−u,…,0,…,u} patients, with u ranging from 0 to 5. Here, 2u represents the maximum imbalance across arms.

### 4.2. Operating Characteristics

The operating characteristics of the different approaches in the uncontrolled setting are reported in Table 1. In scenario 1, the use of the three decision thresholds $C_{n}^{m,a}$, $C_{n}^{m}$, and $\epsilon^{m}$ allowed for desired control of the FWER. The FWER of $C_{n}^{m,a}$ was closer to the prespecified level, with 9.52% of conclusions indicating a promising treatment, where as the FWER was 8.53% for $C_{n}^{m}$ and 9.36% for $\epsilon^{m}$. However, it’s worth noting that the FWER increased to 22.87% for $C_{n}^{s}$ because it was only optimized for a single-arm study. When the number of arms with efficacy and no toxicity increased (scenarios 2 to 5), both $C_{n}^{m}$ and $C_{n}^{m,a}$ exhibited improved power compared to $\epsilon^{m}$. Under the LFC, the empirical power was 72.43% for $C_{n}^{m}$ and 73.22% for $C_{n}^{m,a}$, whereas it was 53.96% with $\epsilon^{m}$ (Table 1). Overall, $C_{n}^{m,a}$ outperformed the other thresholds when there were more than one truly promising arm (scenarios 3, 4, 5, 9, and 10). However, in Scenario 8, where one arm was more effective than $H_{1}$ and two arms were at $H_{0}$, $C_{n}^{m}$ outperformed the other thresholds including $C_{n}^{m,a}$. This is because in the late stages, fewer arms may remain active as the majority of arms are unpromising, resulting in a more stringent threshold for $C_{n}^{m,a}$ in the final analysis. Scenarios involving arms with discordant profiles of efficacy and toxicity (scenarios 6, 7, 10, and 11) resulted in a non-negligible proportion of false positive conclusions. In scenarios 6 and 7, where no arm was identified as promising, the $\epsilon^{m}$ exhibited a lower arm-specific false positive rate compared to other methods for arms with mismatched efficacy and toxicity. The FWERs for $C_{n}^{m,a}$, $C_{n}^{m}$, and $\epsilon^{m}$ were similar in these scenarios and were all lower than that of $C_{n}^{s}$ (52.73% in scenario 6 and 18.52% in scenario 7). In contrast, scenarios 10 and 11, which featured both promising and non-promising arms, saw $C_{n}^{m,a}$ producing slightly higher false positive rates than both $C_{n}^{m}$ and $\epsilon^{m}$.

Lastly, in scenarios 12 and 13, characterized by intermediate probabilities of efficacy and toxicity, $C_{n}^{m,a}$ exhibited a higher rate of false positives compared to $C_{n}^{m}$, while $\epsilon^{m}$ had the lowest proportion of false positives. In general, the higher false positive rate of $C_{n}^{m,a}$ was due to its less stringent threshold. However, its arm-specific Type I error rate remained under 10%.

The operating characteristics of the different approaches in the controlled setting are displayed in Table 2. Similar to the uncontrolled setting, the empirical FWER was controlled at 8.75% for $C_{n}^{m}$, 9.46% for $C_{n}^{m,a}$ and 9.62% for $\epsilon^{m}$. However, it was not controlled for $C_{n}^{s}$ (21.09%). In scenario 2 there was a reduction in power when using the constant $\epsilon^{m}$ compared to $C_{n}^{m}$ (under the LFC, power was 55.52% for $C_{n}^{m}$, 50.01% for $C_{n}^{m,a}$ and 43.73% for $\epsilon^{m}$). Similar results were observed for scenarios 3, 4, 5, 8, and 9. $C_{n}^{m,a}$ outperformed $C_{n}^{m}$ when the three experimental arms were efficacious and not toxic (approximately 66% in each arm in scenario 4, compared to around 55% for $C_{n}^{m}$). Conversely, with only one truly promising arm, $C_{n}^{m}$ exhibited greater power (50.01% vs. 55.52% in scenario 2, arm C). Similarly to uncontrolled settings, scenarios with discordant arms resulted in an increased proportion of false positives (Scenarios 6, 7, 10, and 11). Lastly, in Scenarios 12 and 13, characterized by intermediate probabilities of efficacy and toxicity, $C_{n}^{m,a}$ exhibited a higher rate of false positives than $C_{n}^{m}$ due to the less stringent threshold used, although it still remained controlled at the desired FWER level.

In conclusion, $\epsilon^{m}$ had a higher proportion of early stoppages, while $C_{n}^{m,a}$ resulted in a lower proportion of early stoppages.

**Figure 2.**
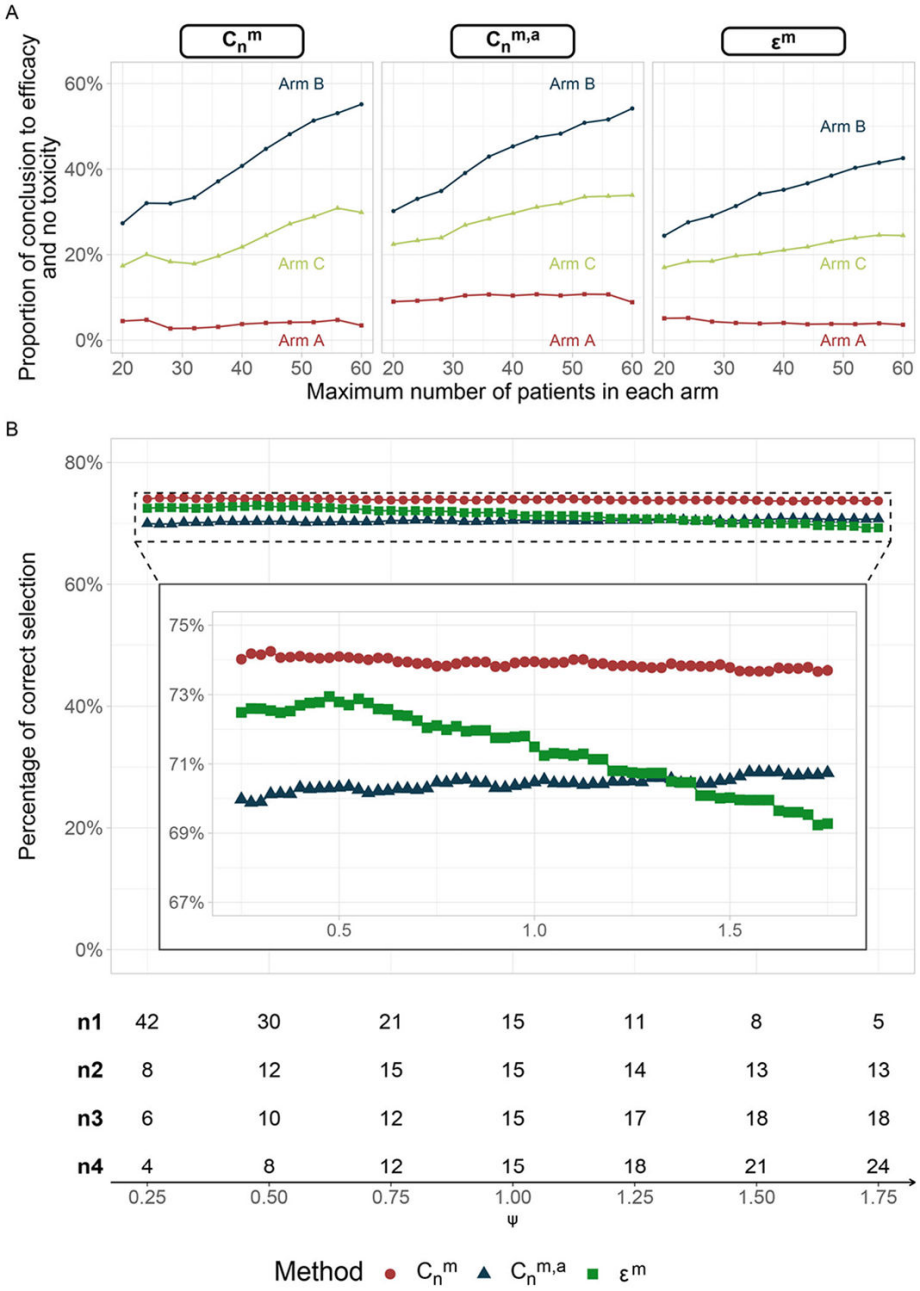
Results for scenario 13 in a controlled setting, calibrated for a maximum sample size of 60 patients per arm. Panel A: percent of conclusions regarding efficacy and absence toxicity in each arm according to the maximum number of enrolled patients. B: proportion of correct selection relative to the accrual rate. The table below the plot represents the additional number of enrolled patients at each interim analysis.

When the sample size was smaller than planned, the estimated FWER remained close to the prespecified level, no more than 12.32%, even with only one third of total expected sample size (20 patients per arm instead of 60, see Supplementary Table S2). Figure 2A depicts the proportion of conclusion of efficacy and acceptable toxicity in each arm under scenario 13 for different sample sizes (see also Supplementary Table S2 in supplementary materials for complete results with lower sample sizes in each arm). Scenario 13 exemplified a situation covering the design hypotheses across the 3 experimental arms (arm A: $H_{0}$, arm B: $H_{1}$, arm C: intermediate efficacy and toxicity). $C_{n}^{m}$, $C_{n}^{m,a}$, and $\epsilon^{m}$ behaved similarly: the proportion of correct selection decreased with a lower sample size while the empirical FWER remained around the specified level. The power and the percentage of correct selection were higher for $C_{n}^{m}$.

When assessing values of ψ ranging from 0.25 to 1.75, the $C_{n}^{m}$ and $C_{n}^{m,a}$ designs had stable operating characteristics with these different recruitment rates (see Figure 2B). In contrast, $\epsilon^{m}$ was more sensitive to variations in accrual rates that deviated from the planned rate. It is also important to note that the percentage of correct selection using $C_{n}^{m,a}$ slightly increased with higher values of ψ. This is because, with larger values of ψ, the sample size at the interim analysis is smaller than expected. Due to the sparser data, the probability of early termination of an arm decreases, allowing for a less stringent cutoff for $C_{n}^{m,a}$. As a result, fewer arms were terminated incorrectly at the interim analysis, leading to an increase in the percentage of correct selection. There was limited impact of interim analyses performed at fixed time points across arms, instead of using a fixed number of patients, on the proportion of conclusion regarding efficacy and acceptable toxicity, as well as on the proportion of correct selections in each arm. The empirical FWER slightly increased when an imbalance appeared across arms (u=1, 2), but it remained stable regardless of the magnitude of imbalance (see Supplementary Table S2).

## 5. The AZA-PLUS Trial

To illustrate the design, we retrospectively planned three interim analyses (at least 20, 40, and 60 patients in each arm) along with the terminal analysis (assuming a maximum of 80 patients in each arm), using the AZA-PLUS trial as an example. We used the following probabilities $\theta_{k}$ for the multinomial distribution of treatment for inefficacy/toxicity to calibrate the design: (0.15, 0.25, 0.15, 0.45), and for efficacy/non toxicity: (0.15, 0.40, 0.05, 0.40). The inefficacy/toxicity hypothesis $H_{0}$ will be used as the prior for the endpoint distributions, and comparisons will be made with the control arm (Azacitidine) for decision rules. Consequently, the prior distribution is Dir(0.15, 0.25, 0.15, 0.45). The optimized parameters for multi-arm $C_{n}^{m}$ were λ=0.63 and γ=1. These parameters controlled the FWER under 15% as planned (actual = 14.84%), and correspondeded to a simulated power of 73.78%.

**Figure 3.**
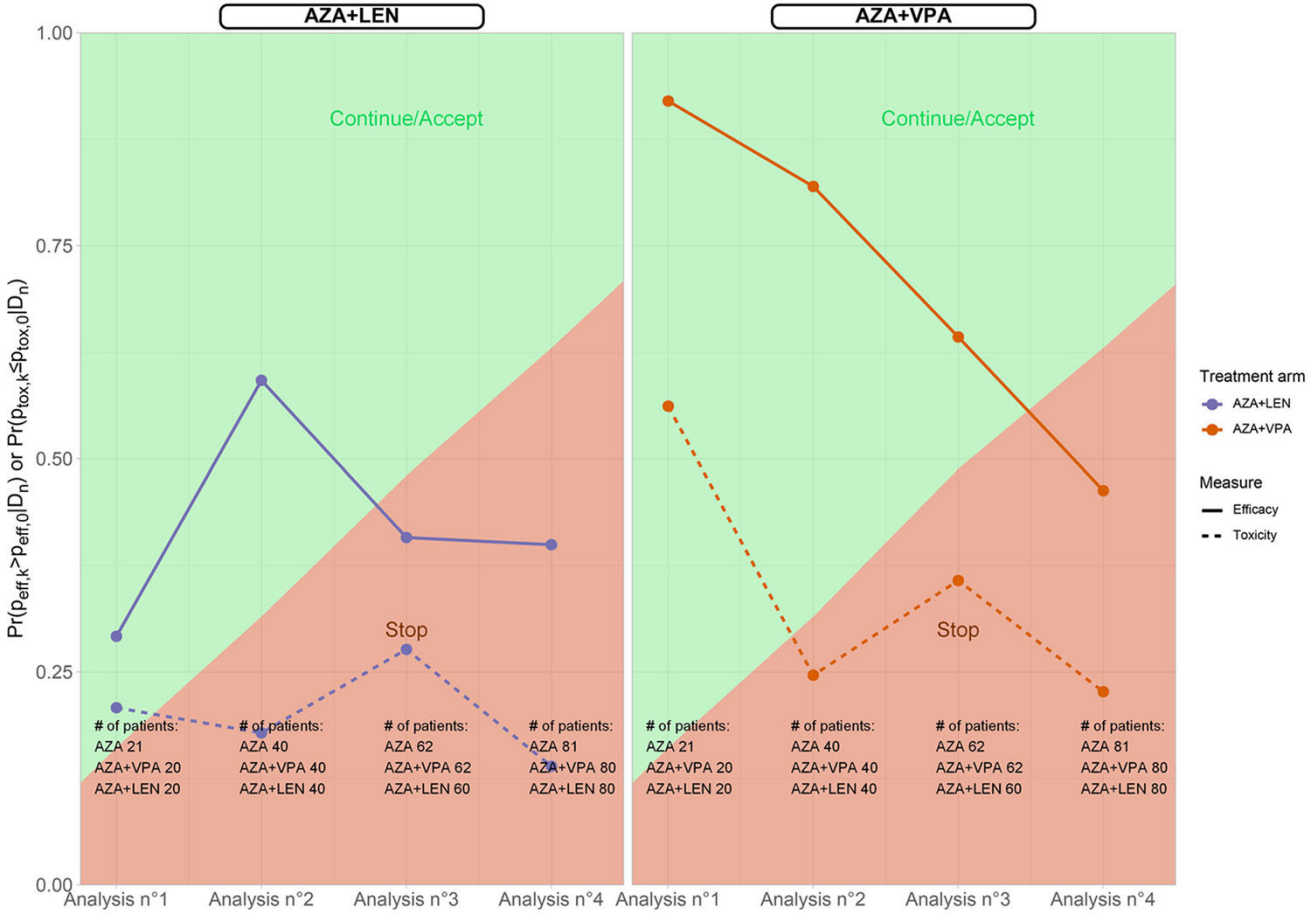
AZA-PLUS trial with the multi-arm $C_{n}^{m}$ threshold: Posterior probabilities of efficacy and no toxicity, along with decision rules, at 3 interim analyses and the final analysis.

The interim analyses had slightly different sample sizes across arms due to randomization. (Figure 3). The retrospective analysis using the proposed design suggested stopping both Azacitidine + Lenalidomide and Azacitidine + Valproic acid arms after the 2nd analysis. Compared to the initial trial, this would have reduced the actual sample size by 120 patients overall (40 patients less in each arm). Of note, given the trial population, the accrual rate was approximately 20 patients per year per arm. Assuming that both efficacy and toxicity criteria would require a follow-up of 6.5 months to eventually take the go/no go decision, the proposed design would have led to an approximate 2-year reduction in trial duration compared to the original design.

## 6. Discussion

We have proposed a Bayesian design to control the FWER in multi-arm multi-stage phase II clinical trials with a joint assessment of efficacy and toxicity. We adapted the BOP2 design (Zhou, Lee, and Yuan 2017) to this setting, using group-sequential decision boundaries that depend on the fraction of the number of enrolled patients, either solely or also accounting for the number of active arms at a given analysis. The proposed decision thresholds demonstrated good operating characteristics with increased power, as shown in single-arm trials, when compared to constant thresholds (Zhou, Lee, and Yuan 2017). This finding is consistent with the work of Jiang et al. (2020), who found that varying boundaries with the course of the study outperformed constant thresholds in terms of power. Additionally, their approach used separate constant thresholds for efficacy and toxicity, which sometimes may result in thresholds that are more stringent for one endpoint than the other. It might be beneficial to consider additional constraints for these constant thresholds.

Two functions were defined for the decision boundaries, with the one depending on the number of active arms at the time of interim analyses outperforming the one depending only on the fraction of included patients overall. Ensuring less stringent boundaries when there remained some unpromising arms ensured greater power for truly promising arms. However, this came at the cost of a slightly increased false positive rate in trials that included truly promising arms alongside ineffective or toxic ones.

The design appeared robust in the face of departures from the planned setting, including variations in maximum sample size, accrual rate, and balance across arms at each analysis. The design however encountered challenges when dealing a drug exhibiting a discordant profile of efficacy and toxicity (i.e., efficacious but toxic or inefficacious but safe). This could be attributed to the computation of the decision thresholds, which are optimized to control the FWER under the global null hypothesis, where all treatments are assumed to be inefficacious and toxic. These situations may arise when evaluating the association of treatments that could potentially lead to antagonistic interactions, for example. Additionally, the method demonstrated robustness in terms of the choice of prior. The selection of a pessimistic prior, as in original BOP2 design, was made to ensure that interim analysis conclusions are robust. A change in prior with a small effective sample size does not alter the results significantly. However, one should exercise caution when considering the weight of the prior relative to interim analysis results. Nevertheless, through the calibration process, the influence of prior distributions can be further diminished. Similar to the BOP2 design, the proposed thresholds are optimized considering the entire distribution of efficacy and toxicity, thereby taking into account the correlation between efficacy and toxicity. This approach provides adaptability, allowing for the adjustment of the correlation between efficacy and toxicity. However, one should exercise caution when choosing this correlation, as an overestimated correlation may result in a slight inflation of the FWER (results not shown).

Future research directions can be explored in conjunction with the proposed method. Jiang et al. (2021) recently proposed a seamless phase I/II design in which patients are split into indication-specific parallel subgroups for the phase II part of the design, similar to an uncontrolled basket trial. They rely on Bayesian hierarchical modeling to borrow information across subgroups. Further evaluation is needed to quantify the benefit of such an approach in our controlled setting. Furthermore, while we have only implemented futility stopping rules, efficacy stopping rules could also be considered for the multi-arm multi-stage trial, where a promising treatment showing signals of efficacy without toxicity could graduate early (Blenkinsop, Parmar, and Choodari-Oskooei 2019). Of note, the proposed method could be considered as a selection design with multiple treatment candidates for a common indication. Since there is no strong consensus on Type I and II error rates for phase II trials, emphasis should be made on FWER or power relatively to the purpose and settings of the study (Stallard 2012), compromising somewhat on the risk of false positives and false negatives, prior to formal efficacy assessment in phase III.

In conclusion, the proposed design for multi-arm multi-stage trials has demonstrated promising operating characteristics and could be employed to screen multiple treatments in phase II trials. Accounting for the available fraction of information and for the number of active arms allowed for an improvement in power, particularly in situations with multiple promising treatments. The R package to implement the methods proposed in this article is available at https://github.com/GuillaumeMulier/multibrasBOP2.

## Supplementary Material

Supp 1

## Figures and Tables

**Table 1.** Operating characteristics for each arm under the 13 scenarios (Sc) for the uncontrolled designs: Family-Wise Error Rate (FWER), percent of conclusion of efficacy and no toxicity (ENT), percent of early stopping (ES), and mean sample size (SS).

		$C_{n}^{m}$			$C_{n}^{m,a}$			$\epsilon^{m}$		
Sc	Arm	*ENT*	*ES*	*SS*	*ENT*	*ES*	*SS*	*ENT*	*ES*	*SS*
1			FWER: 8.53%			FWER: 9.52%			FWER: 9.36%	
	A(0.45, 0.30)	2.96	89.76	31.03	3.54	82.14	35.72	3.15	95.51	19.26
	B(0.45, 0.30)	2.78	89.69	30.80	3.40	82.30	35.57	3.31	95.27	19.27
	C(0.45, 0.30)	3.09	89.33	31.03	3.76	82.32	35.59	3.25	95.32	19.24
2	A(0.60, 0.20)	72.43	19.51	54.63	73.22	13.73	56.54	53.96	45.37	41.07
	B(0.45, 0.30)	2.92	89.67	30.91	5.66	78.05	36.69	3.20	95.34	19.20
	C(0.45, 0.30)	3.12	89.48	30.89	5.90	77.97	36.70	3.34	95.28	19.30
3	A(0.60, 0.20)	72.43	19.51	54.63	79.96	11.45	56.99	53.96	45.37	41.07
	B(0.60, 0.20)	72.89	18.99	54.74	80.64	10.68	57.20	54.20	45.15	41.10
	C(0.45, 0.30)	2.85	89.30	31.10	9.00	70.62	38.46	3.10	95.54	19.19
4	A(0.60, 0.20)	72.43	19.51	54.63	86.06	7.47	57.74	53.96	45.37	41.07
	B(0.60, 0.20)	72.89	18.99	54.74	86.11	7.27	57.85	54.20	45.15	41.10
	C(0.60, 0.20)	72.50	19.28	54.62	85.19	7.67	57.68	53.36	46.00	40.80
5	A(0.65, 0.20)	80.67	13.95	56.20	88.91	5.92	58.12	68.50	30.98	46.76
	B(0.65, 0.20)	80.33	14.40	56.12	88.71	5.97	58.15	68.44	31.30	46.68
	C(0.65, 0.20)	80.68	13.74	56.23	89.06	5.87	58.14	68.77	30.82	46.85
6			FWER: 31.25%			FWER: 34.42%			FWER: 31.43%	
	A(0.60, 0.30)	18.44	67.79	40.55	18.97	58.28	43.68	22.43	74.19	28.95
	B(0.45, 0.20)	13.06	71.57	38.30	18.34	58.34	44.08	8.86	89.25	23.27
	C(0.45, 0.30)	3.14	89.51	31.00	5.39	78.36	36.64	3.19	95.33	19.28
7			FWER: 7.49%			FWER: 8.22%			FWER: 8.88%	
	A(0.70, 0.40)	0.98	96.17	27.07	0.74	94.14	28.32	3.48	93.41	20.70
	B(0.40, 0.10)	3.86	87.05	31.49	5.02	81.58	36.26	2.65	96.02	19.82
	C(0.45, 0.30)	2.86	89.56	31.14	3.07	84.41	35.04	3.06	95.59	19.23
8	A(0.65, 0.20)	80.67	13.95	56.20	79.37	10.70	57.23	68.50	30.98	46.76
	B(0.45, 0.30)	3.05	89.22	31.03	6.35	77.82	36.63	3.18	95.57	19.17
	C(0.45, 0.30)	2.92	89.44	30.96	5.97	77.30	36.84	3.08	95.55	19.20
9	A(0.65, 0.20)	80.67	13.95	56.20	88.69	5.96	58.12	68.50	30.98	46.76
	B(0.60, 0.20)	72.89	19.14	54.68	85.87	7.67	57.63	55.09	44.45	41.45
	C(0.60, 0.20)	72.36	19.76	54.44	85.38	7.81	57.61	53.18	46.13	40.72
10	A(0.60, 0.20)	72.43	19.51	54.63	80.17	11.48	56.99	53.96	45.37	41.07
	B(0.60, 0.20)	72.89	18.99	54.74	80.42	11.17	57.10	54.20	45.15	41.10
	C(0.70, 0.35)	5.36	85.88	33.58	9.60	70.52	37.76	12.93	81.35	26.58
11	A(0.60, 0.20)	72.43	19.51	54.63	82.16	9.57	57.33	53.96	45.37	41.07
	B(0.60, 0.20)	72.89	18.99	54.74	82.75	8.98	57.52	54.20	45.15	41.10
	C(0.45, 0.20)	12.30	72.83	37.86	27.17	49.15	46.01	8.06	89.83	22.79
12	A(0.60, 0.20)	72.43	19.51	54.63	79.94	10.51	57.15	53.96	45.37	41.07
	B(0.50, 0.25)	19.32	65.03	41.09	33.12	42.81	47.85	14.89	83.30	25.35
	C(0.50, 0.25)	18.97	64.65	41.25	32.76	42.70	48.06	14.98	83.01	25.39
13	A(0.45, 0.30)	2.96	89.76	31.03	7.31	74.29	37.63	3.15	95.51	19.26
	B(0.60, 0.20)	72.82	19.01	54.76	77.08	11.94	56.95	54.02	45.35	41.04
	C(0.50, 0.25)	19.23	64.86	41.03	28.89	46.73	47.14	14.48	83.40	25.36

NOTE: True probabilities of efficacy and toxicity in each arm are given in parentheses: (Efficacy, Toxicity).

**Table 2.** Operating characteristics for each experimental (Exp.) arm under the 13 scenarios (Sc) for the controlled designs: Family-Wise Error Rate (FWER), percent of conclusion of efficacy and no toxicity (ENT), percent of early stopping (ES), and mean sample size (SS).

	Controlled	$C_{n}^{m}$			$C_{n}^{m,a}$			$\epsilon^{m}$		
Sc	Exp. Arm	*ENT*	*ES*	*SS*	*ENT*	*ES*	*SS*	*ENT*	*ES*	*SS*
1			FWER: 8.75%			FWER: 9.46%			FWER: 9.62%	
	A(0.60, 0.40)	3.44	86.56	32.13	4.62	79.98	37.00	3.60	94.72	20.14
	B(0.60, 0.40)	3.31	86.33	32.04	4.52	80.01	36.78	3.96	94.53	20.10
	C(0.60, 0.40)	3.27	87.56	31.64	4.66	80.73	36.64	3.66	94.71	20.15
2	A(0.75, 0.30)	55.52	32.44	50.54	50.01	29.94	52.58	43.73	54.62	37.65
	B(0.60, 0.40)	3.72	86.49	32.23	6.51	76.28	37.78	3.86	94.67	20.23
	C(0.60, 0.40)	3.70	86.52	31.98	6.59	76.63	37.67	3.97	94.54	20.16
3	A(0.75, 0.30)	55.52	32.44	50.54	56.60	25.57	53.39	43.73	54.62	37.65
	B(0.75, 0.30)	54.85	33.03	50.37	55.90	26.29	53.22	42.26	56.11	37.16
	C(0.60, 0.40)	3.55	86.74	31.87	9.37	72.68	38.42	3.83	94.79	20.12
4	A(0.75, 0.30)	55.52	32.44	50.54	66.66	20.49	54.31	43.73	54.62	37.65
	B(0.75, 0.30)	54.85	33.03	50.37	66.06	21.12	54.16	42.26	56.11	37.16
	C(0.75, 0.30)	55.50	32.96	50.42	66.71	20.85	54.24	43.13	55.38	37.56
5	A(0.80, 0.25)	80.09	14.57	55.61	88.66	7.40	57.85	65.02	34.29	45.71
	B(0.80, 0.25)	79.74	15.32	55.38	88.09	7.97	57.62	65.47	33.86	45.95
	C(0.80, 0.25)	79.46	15.12	55.39	88.21	7.53	57.74	65.14	34.17	45.82
6			FWER: 31.71%			FWER: 30.30%			FWER: 30.46%	
	A(0.60, 0.30)	19.83	63.48	40.76	20.44	56.76	44.70	21.63	75.05	29.67
	B(0.75, 0.40)	14.47	69.95	38.74	15.76	62.19	43.10	11.06	86.61	24.41
	C(0.60, 0.40)	3.15	86.85	31.94	6.21	76.95	37.48	3.95	94.51	20.12
7			FWER: 14.51%			FWER: 13.02%			FWER: 18.78%	
	A(0.50, 0.25)	9.87	76.23	36.26	9.19	73.03	39.61	14.93	80.85	27.68
	B(0.80, 0.45)	2.28	90.46	29.76	2.63	86.89	33.71	1.74	96.94	18.86
	C(0.60, 0.40)	3.49	86.65	32.16	3.77	82.00	36.60	3.82	94.76	20.14
8	A(0.80, 0.25)	80.09	14.57	55.61	75.23	13.48	56.73	65.02	34.29	45.71
	B(0.60, 0.40)	3.19	86.77	32.00	6.50	76.69	37.73	3.83	94.50	20.22
	C(0.60, 0.40)	3.48	86.74	32.10	6.17	77.01	37.63	3.58	94.68	20.07
9	A(0.80, 0.25)	80.09	14.57	55.61	85.46	8.76	57.61	65.02	34.29	45.71
	B(0.75, 0.30)	54.71	33.43	50.18	68.36	20.53	54.27	42.94	55.56	37.38
	C(0.75, 0.30)	54.97	32.61	50.50	68.48	19.94	54.42	43.08	55.45	37.41
10	A(0.75, 0.30)	55.52	32.44	50.54	58.15	24.91	53.53	43.73	54.62	37.65
	B(0.75, 0.30)	54.85	33.03	50.37	57.04	25.59	53.36	42.26	56.11	37.16
	C(0.75, 0.45)	8.87	78.16	35.42	15.97	64.85	40.92	12.14	84.29	25.95
11	A(0.75, 0.30)	55.52	32.44	50.54	55.69	26.32	53.23	43.73	54.62	37.65
	B(0.75, 0.30)	54.85	33.03	50.37	54.67	26.97	53.07	42.26	56.11	37.16
	C(0.50, 0.25)	2.23	90.61	29.73	4.94	81.08	34.99	2.01	96.82	18.88
12	A(0.75, 0.30)	55.52	32.44	50.54	60.66	23.50	53.79	43.73	54.62	37.65
	B(0.70, 0.35)	29.90	54.18	43.82	40.31	39.91	48.98	24.34	73.17	30.25
	C(0.70, 0.35)	29.94	53.56	44.12	40.62	39.72	49.12	25.06	72.44	30.63
13	A(0.60, 0.40)	3.44	86.56	32.13	8.87	73.10	38.47	3.60	94.72	20.14
	B(0.75, 0.30)	55.15	32.77	50.42	54.17	26.99	53.13	42.55	55.61	37.27
	C(0.70, 0.35)	29.86	54.35	43.75	33.89	43.98	48.15	24.47	73.24	30.26

NOTE: True probabilities of efficacy and toxicity in each experimental arm are given in parentheses: (Efficacy, Toxicity), where the values of the control arm are (0.60, 0.40).
