# Supplementary material for: Bayesian Optimal Designs for Multi-Arm Multi-Stage Phase II Randomized Clinical Trials with Multiple Endpoints
Source: Stat Biopharm Res. Author manuscript; Available in PMC 2024 Sep 19. (PMC11412438; doi:10.1080/19466315.2024.2344543)
Supplement: Supp 1 [file NIHMS1992888-supplement-Supp_1.pdf]

Supplementary Materials for Bayesian optimal designs for multi-arm multi-stage phase II randomized clinical trials with multiple endpoints

1 Scenarios

Table S1: Simulation scenarios for uncontrolled and controlled settings: probabilities of the four outcomes ($P(Y_E = 1 \cap Y_T = 1)$,$P(Y_E = 1 \cap Y_T = 0)$, $P(Y_E = 0 \cap Y_T = 1)$, $P(Y_E = 0 \cap Y_T = 0)$).

Uncontrolled setting	Arm A	Arm B	Arm C
Sc1: Global H_0	(0.15, 0.30, 0.15, 0.40)	(0.15, 0.30, 0.15, 0.40)	(0.15, 0.30, 0.15, 0.40)
Sc2: LFC	(0.18, 0.42, 0.02, 0.38)	(0.15, 0.30, 0.15, 0.40)	(0.15, 0.30, 0.15, 0.40)
Sc3: A+B H_1, C H_0	(0.18, 0.42, 0.02, 0.38)	(0.18, 0.42, 0.02, 0.38)	(0.15, 0.30, 0.15, 0.40)
Sc4: A+B+C H_1	(0.18, 0.42, 0.02, 0.38)	(0.18, 0.42, 0.02, 0.38)	(0.18, 0.42, 0.02, 0.38)
Sc5: A+B+C better than H_1	(0.15, 0.50, 0.05, 0.30)	(0.15, 0.50, 0.05, 0.30)	(0.15, 0.50, 0.05, 0.30)
Sc6: A ineffective, B toxic, C H_0	(0.20, 0.40, 0.10, 0.30)	(0.10, 0.35 ,0.10 ,0.45)	(0.15, 0.30, 0.15, 0.40)
Sc7: A ineffective, B toxic, C H_0 (2)	(0.20, 0.50, 0.20, 0.10)	(0.05, 0.35 ,0.05 ,0.55)	(0.15, 0.30, 0.15, 0.40)
Sc8: A better than H_1, B+C H_0	(0.15, 0.50, 0.05, 0.30)	(0.15, 0.30, 0.15, 0.40)	(0.15, 0.30, 0.15, 0.40)
Sc9: A better than H_1, B+C H_1	(0.15, 0.50, 0.05, 0.30)	(0.18, 0.42, 0.02, 0.38)	(0.18, 0.42, 0.02, 0.38)
Sc10: A+B H_1, C toxic	(0.18, 0.42, 0.02, 0.38)	(0.18, 0.42, 0.02, 0.38)	(0.20, 0.50, 0.15, 0.15)
Sc11: A+B H_1, C ineffective	(0.18, 0.42, 0.02, 0.38)	(0.18, 0.42, 0.02, 0.38)	(0.10, 0.35, 0.10, 0.45)
Sc12: A H_1, B+C intermediate	(0.18, 0.42, 0.02, 0.38)	(0.15, 0.35, 0.10, 0.40)	(0.15, 0.35, 0.10, 0.40)
Sc13: A H_0, B H_1, C intermediate	(0.15, 0.30, 0.15, 0.40)	(0.18, 0.42, 0.02, 0.38)	(0.15, 0.35, 0.10, 0.40)
Controlled setting			
Control arm (0.30, 0.30, 0.10, 0.30)	Arm A	Arm B	Arm C
Sc1: Global H_0	(0.30, 0.30, 0.10, 0.30)	(0.30, 0.30, 0.10, 0.30)	(0.30, 0.30, 0.10, 0.30)
Sc2: LFC	(0.25, 0.50, 0.05, 0.20)	(0.30, 0.30, 0.10, 0.30)	(0.30, 0.30, 0.10, 0.30)
Sc3: A+B H_1, C H_0	(0.25, 0.50, 0.05, 0.20)	(0.25, 0.50, 0.05, 0.20)	(0.30, 0.30, 0.10, 0.30)
Sc4: A+B+C H_1	(0.25, 0.50, 0.05, 0.20)	(0.25, 0.50, 0.05, 0.20)	(0.25, 0.50, 0.05, 0.20)
Sc5: A+B+C better than H_1	(0.20, 0.60, 0.05, 0.15)	(0.20, 0.60, 0.05, 0.15)	(0.20, 0.60, 0.05, 0.15)
Sc6: A ineffective, B toxic, C H_0	(0.25, 0.50, 0.15, 0.10)	(0.20, 0.40 ,0.10 ,0.30)	(0.30, 0.30, 0.10, 0.30)
Sc7: A ineffective, B toxic, C H_0 (2)	(0.30, 0.50, 0.15, 0.05)	(0.20, 0.30 ,0.05 ,0.45)	(0.30, 0.30, 0.10, 0.30)
Sc8: A better than H_1, B+C H_0	(0.20, 0.60, 0.05, 0.15)	(0.30, 0.30, 0.10, 0.30)	(0.30, 0.30, 0.10, 0.30)
Sc9: A better than H_1, B+C H_1	(0.20, 0.60, 0.05, 0.15)	(0.25, 0.50, 0.05, 0.20)	(0.25, 0.50, 0.05, 0.20)
Sc10: A+B H_1, C toxic	(0.25, 0.50, 0.05, 0.20)	(0.25, 0.50, 0.05, 0.20)	(0.25, 0.50, 0.20, 0.05)
Sc11: A+B H_1, C ineffective	(0.25, 0.50, 0.05, 0.20)	(0.25, 0.50, 0.05, 0.20)	(0.20, 0.30, 0.05, 0.45)
Sc12: A H_1, B+C intermediate	(0.25, 0.50, 0.05, 0.20)	(0.20, 0.50, 0.15, 0.15)	(0.20, 0.50, 0.15, 0.15)
Sc13: A H_0, B H_1, C intermediate	(0.30, 0.30, 0.10, 0.30)	(0.25, 0.50, 0.05, 0.20)	(0.20, 0.50, 0.15, 0.15)

2 Additional results for controlled design

Table S2: Scenario 13: Percentage of correct selection (PCS) and estimated FWER (Family-Wise Error Rate) with a lower than expected accrual ("Maximum sample size in each arm") given a planned total sample size of $n = 60$, or with an imbalance at each interim analysis in the number of patients across arms ("Maximum imbalance").

Variable	Value	C_n^m		$C_n^{m,a}$		ϵ^m	
		FWER	PCS	FWER	PCS	FWER	PCS
Maximum sample size in each arm	20	10.62	68.50	11.61	66.25	12.00	67.44
	24	11.49	69.08	10.41	66.84	12.32	68.00
	28	7.54	70.30	10.37	67.13	10.84	68.74
	32	7.59	70.90	11.74	67.24	10.76	69.20
	36	8.45	71.46	11.99	67.96	10.27	70.04
	40	9.80	71.73	12.22	68.41	10.63	70.03
	44	10.11	72.07	10.87	68.53	10.21	70.38
	48	10.94	72.27	11.00	68.62	10.14	70.56
	52	11.13	72.75	10.73	68.86	10.23	70.88
	56	11.50	72.49	10.37	69.08	9.79	71.00
	60	8.75	73.95	9.46	70.47	9.62	71.49
Maximum imbalance	0	9.32	74.12	10.09	70.76	10.03	71.54
	1	9.81	73.72	11.15	70.13	10.38	71.36
	2	9.57	73.71	11.19	70.10	10.74	71.39
	3	9.88	73.61	11.45	70.05	10.75	71.16
	4	10.18	73.77	11.64	70.09	10.61	71.31
	5	10.10	73.45	11.61	69.75	10.48	71.11

Table S3: Operating characteristics for each arm under the 13 scenarios (Sc) for the controlled C_n^s: Family-Wise Error Rate (FWER), percent of conclusion to efficacy and no toxicity (ENT), percent of early stopping (ES) and mean sample size (SS). In parentheses are provided the probability of efficacy and toxicity in each experimental arm (Efficacy, Toxicity) given a control arm at (0.60, 0.40).

Sc	Arm	ENT	ES	SS
1		FWER: 21.09%		
	A (0.60,0.40)	8.89	79.27	34.69
	B (0.60,0.40)	9.10	79.48	34.47
	C (0.60,0.40)	8.54	80.44	34.11
2	A (0.75,0.30)	68.27	25.15	52.27
	B (0.60,0.40)	8.87	79.25	34.77
	C (0.60,0.40)	9.01	79.89	34.48
3	A (0.75,0.30)	68.27	25.15	52.27
	B (0.75,0.30)	67.71	26.23	52.01
	C (0.60,0.40)	8.75	80.10	34.38
4	A (0.75,0.30)	68.27	25.15	52.27
	B (0.75,0.30)	67.71	26.23	52.01
	C (0.75,0.30)	67.77	25.79	52.13
5	A (0.80,0.25)	87.26	10.71	56.60
	B (0.80,0.25)	86.48	11.28	56.34
	C (0.80,0.25)	86.40	11.18	56.38
6	A (0.60,0.30)	30.98	54.90	43.24
	B (0.75,0.40)	25.39	61.16	41.27
	C (0.60,0.40)	8.58	80.00	34.38
7	A (0.50,0.25)	17.62	68.80	38.60
	B (0.80,0.45)	5.24	85.64	31.72
	C (0.60,0.40)	8.77	79.77	34.62
8	A (0.80,0.25)	87.26	10.71	56.60
	B (0.60,0.40)	8.80	79.81	34.52
	C (0.60,0.40)	8.61	79.60	34.52
9	A (0.80,0.25)	87.26	10.71	56.60
	B (0.75,0.30)	67.10	26.37	51.89
	C (0.75,0.30)	68.00	25.52	52.25
10	A (0.75,0.30)	68.27	25.15	52.27
	B (0.75,0.30)	67.71	26.23	52.01
	C (0.75,0.45)	15.68	71.47	37.69
11	A (0.75,0.30)	68.27	25.15	52.27
	B (0.75,0.30)	67.71	26.23	52.01
	C (0.50,0.25)	5.29	85.79	31.70
12	A (0.75,0.30)	68.27	25.15	52.27
	B (0.70,0.35)	43.44	45.25	46.24
	C (0.70,0.35)	43.97	44.49	46.53
13	A (0.60,0.40)	8.89	79.27	34.69
	B (0.75,0.30)	68.07	25.42	52.28
	C (0.70,0.35)	43.70	45.46	46.12

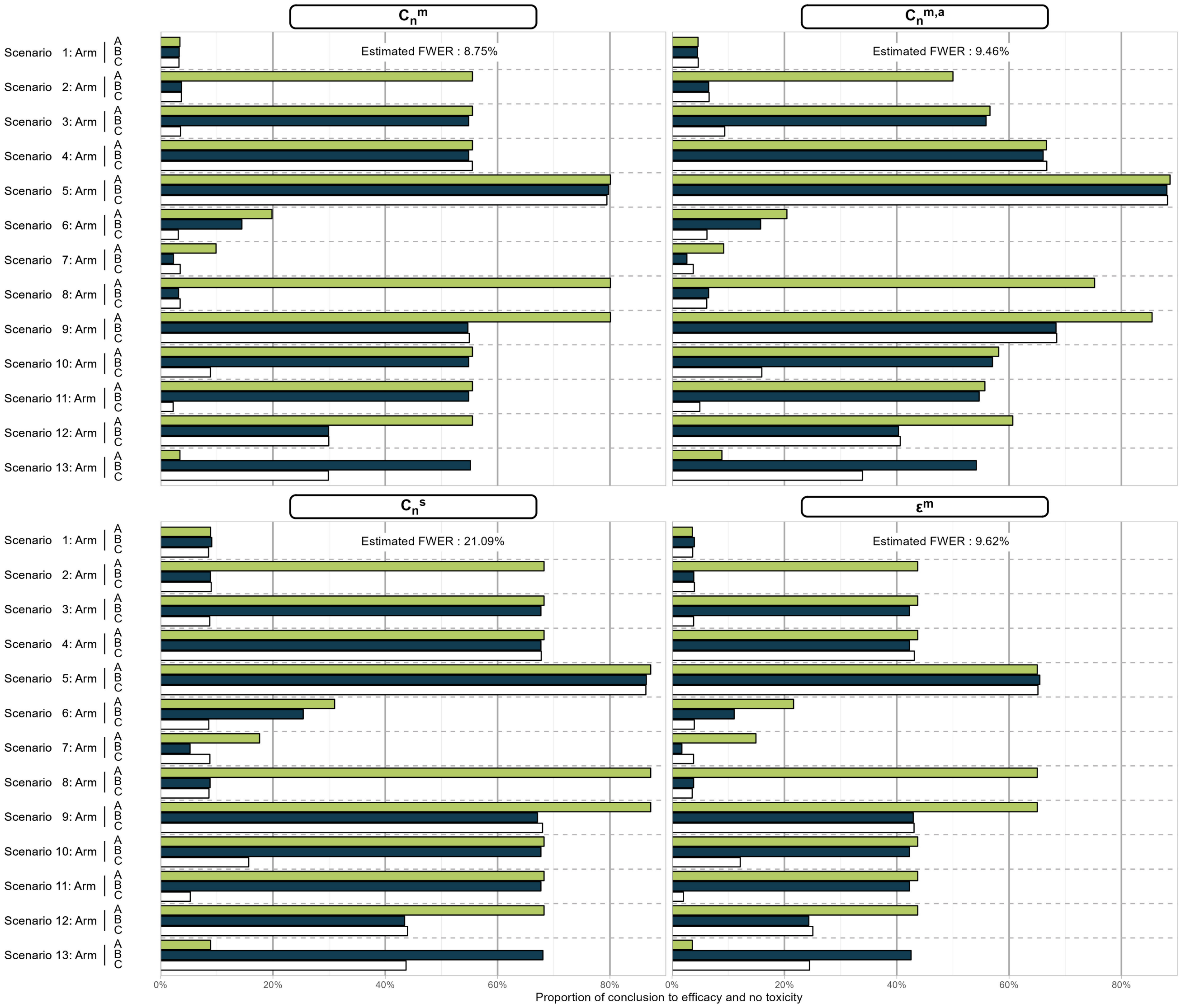

Figure S1: Proportion of conclusion of efficacy and no toxicity in simulated trials for the controlled design.

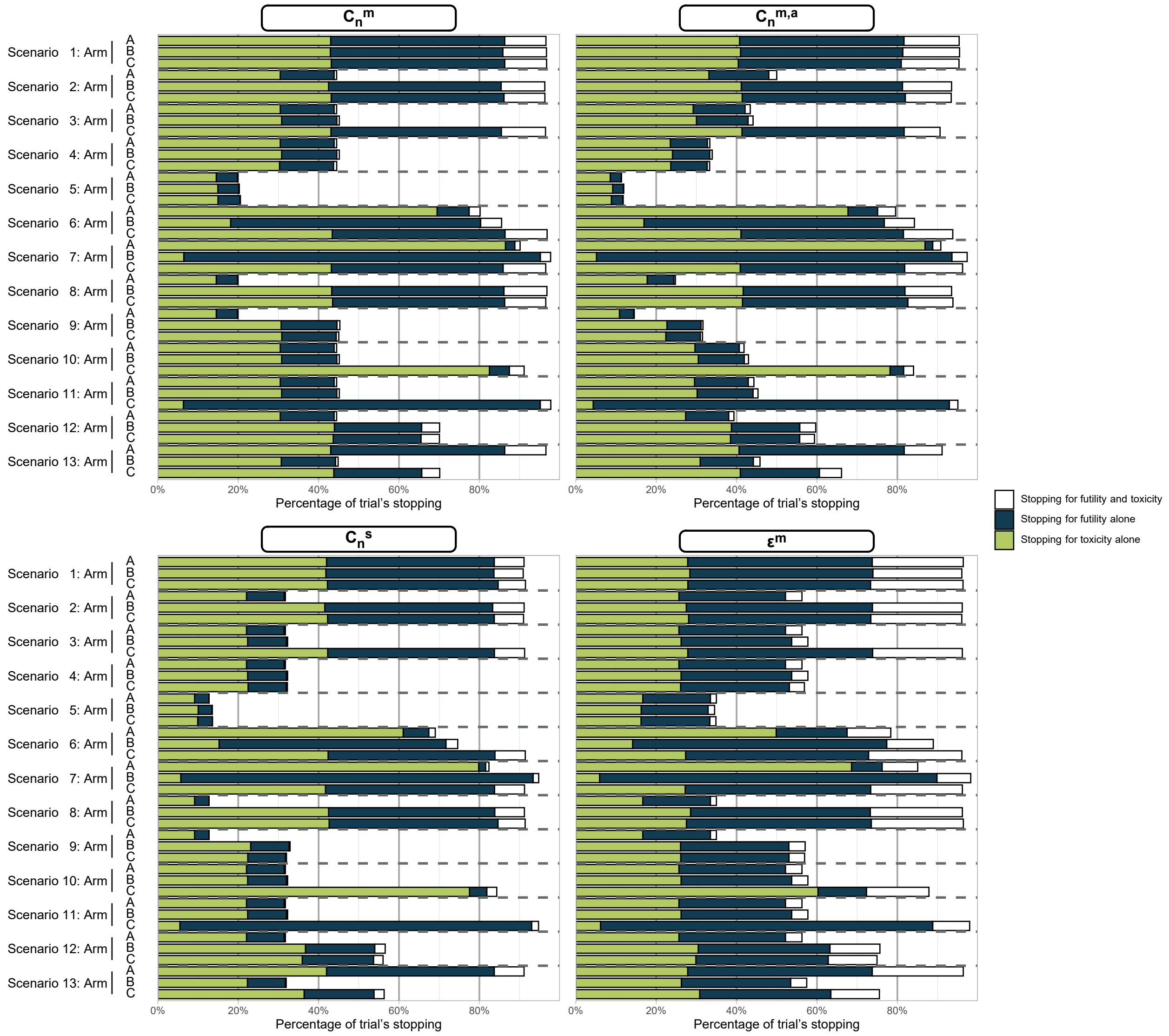

Figure S2: Proportion of stopping for futility and/or toxicity in simulated trials for the controlled design.

3 Additional results for uncontrolled setting

Table S4: Operating characteristics for each arm under the 13 scenarios (Sc) for the uncontrolled C_n^s: Family-Wise Error Rate (FWER), percent of conclusion to efficacy and no toxicity (ENT), percent of early stopping (ES) and mean sample size (SS). In parentheses are provided the probability of efficacy and toxicity in each experimental arm (Efficacy, Toxicity).

Sc	Arm	ENT	ES	SS
1		FWER: 22.87%		
	A(0.45,0.30)	8.20	80.91	35.84
	B(0.45,0.30)	8.07	80.38	35.64
	C(0.45,0.30)	8.56	80.55	35.77
2	A(0.60,0.20)	84.85	10.59	57.13
	B(0.45,0.30)	8.37	80.44	35.69
	C(0.45,0.30)	8.61	80.15	35.80
3	A(0.60,0.20)	84.85	10.59	57.13
	B(0.60,0.20)	85.22	10.48	57.19
	C(0.45,0.30)	8.64	80.09	35.99
4	A(0.60,0.20)	84.85	10.59	57.13
	B(0.60,0.20)	85.22	10.48	57.19
	C(0.60,0.20)	84.68	10.54	57.08
5	A(0.65,0.20)	88.53	7.88	57.82
	B(0.65,0.20)	88.55	7.81	57.86
	C(0.65,0.20)	88.81	7.49	57.89
6	A(0.60,0.30)	29.19	54.27	44.79
	B(0.45,0.20)	26.44	59.92	43.03
	C(0.45,0.30)	8.57	80.61	35.75
7	A(0.70,0.40)	1.91	92.51	29.24
	B(0.40,0.10)	9.58	80.34	35.80
	C(0.45,0.30)	8.38	80.04	35.91
8	A(0.65,0.20)	88.53	7.88	57.82
	B(0.45,0.30)	8.79	80.60	35.68
	C(0.45,0.30)	8.58	80.19	35.92
9	A(0.65,0.20)	88.53	7.88	57.82
	B(0.60,0.20)	85.05	10.39	57.10
	C(0.60,0.20)	84.56	10.76	57.05
10	A(0.60,0.20)	84.85	10.59	57.13
	B(0.60,0.20)	85.22	10.48	57.19
	C(0.70,0.35)	9.58	77.39	36.81
11	A(0.60,0.20)	84.85	10.59	57.13
	B(0.60,0.20)	85.22	10.48	57.19
	C(0.45,0.20)	25.57	60.88	42.77
12	A(0.60,0.20)	84.85	10.59	57.13
	B(0.50,0.25)	35.18	49.98	46.14
	C(0.50,0.25)	35.63	49.54	46.34
13	A(0.45,0.30)	8.20	80.91	35.84
	B(0.60,0.20)	85.31	10.33	57.23
	C(0.50,0.25)	35.93	49.84	46.24

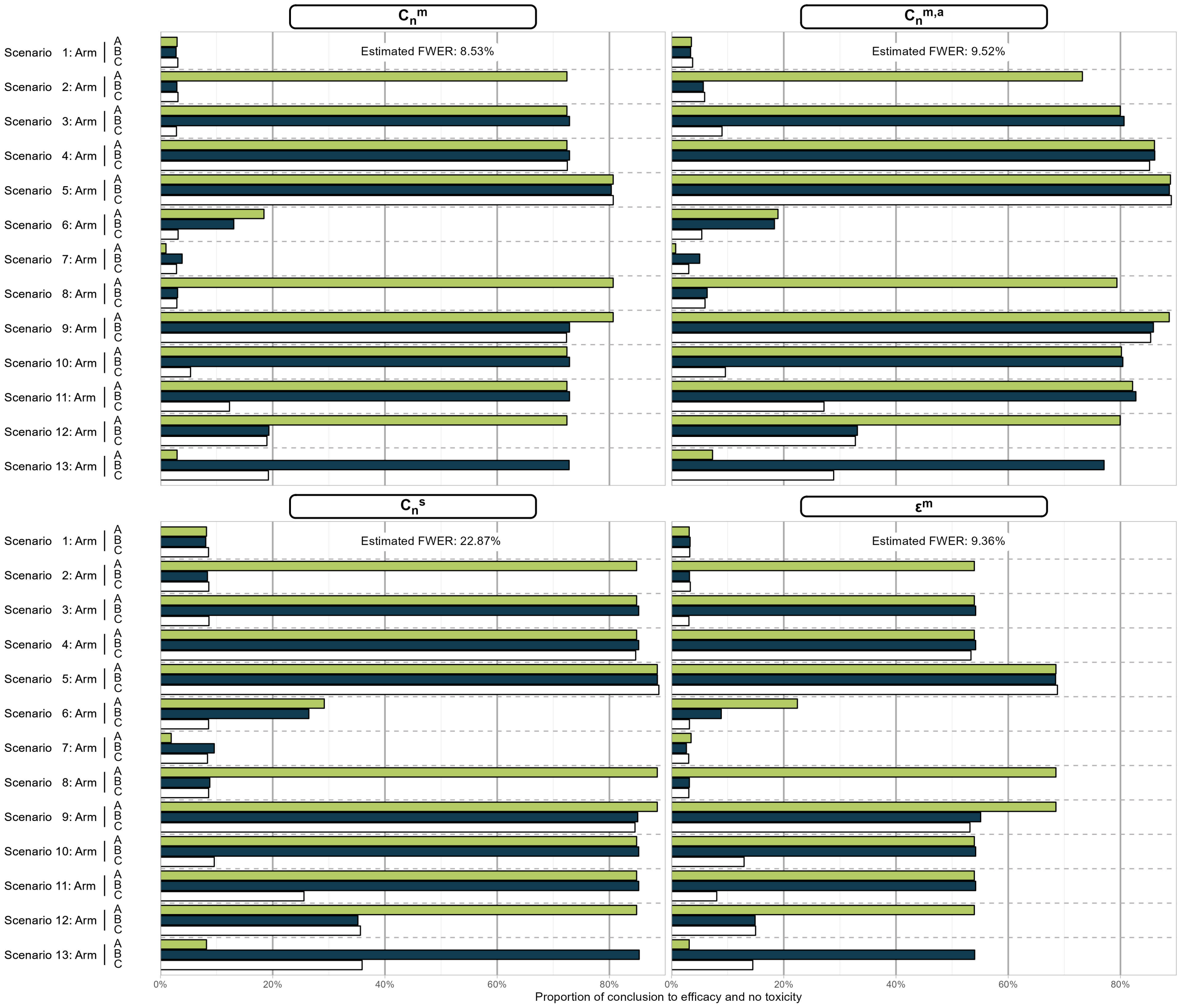

Figure S3: Proportion of conclusion of efficacy and no toxicity in simulated trials for the uncontrolled design.
